# C-Type Natriuretic Peptide Ameliorates Vascular Injury and Improves Neurological Outcomes in Neonatal Hypoxic-Ischemic Brain Injury in Mice

**DOI:** 10.3390/ijms22168966

**Published:** 2021-08-20

**Authors:** Guofang Shen, Shirley Hu, Zhen Zhao, Lubo Zhang, Qingyi Ma

**Affiliations:** 1 The Lawrence D. Longo, MD Center for Perinatal Biology, Department of Basic Sciences, Loma Linda University School of Medicine, Loma Linda, CA 92350, USA; gshen@llu.edu (G.S.); Shu@llu.edu (S.H.); lzhang@llu.edu (L.Z.); 2 Center for Neurodegeneration and Regeneration, Zilkha Neurogenetic Institute and Department of Physiology and Neuroscience, Keck School of Medicine, University of Southern California, Los Angeles, CA 90033, USA; zzhao@usc.edu

**Keywords:** hypoxic-ischemic encephalopathy, C-type natriuretic peptide, vascular protection, endothelial cells, NPR2, NPR3

## Abstract

C-type natriuretic peptide (CNP) is an important vascular regulator that is present in the brain. Our previous study demonstrated the innate neuroprotectant role of CNP in the neonatal brain after hypoxic-ischemic (HI) insults. In this study, we further explored the role of CNP in cerebrovascular pathology using both in vivo and in vitro models. In a neonatal mouse HI brain injury model, we found that intracerebroventricular administration of recombinant CNP dose-dependently reduces brain infarct size. CNP significantly decreases brain edema and immunoglobulin G (IgG) extravasation into the brain tissue, suggesting a vasculoprotective effect of CNP. Moreover, in primary brain microvascular endothelial cells (BMECs), CNP dose-dependently protects BMEC survival and monolayer integrity against oxygen-glucose deprivation (OGD). The vasculoprotective effect of CNP is mediated by its innate receptors NPR2 and NPR3, in that inhibition of either NPR2 or NPR3 counteracts the protective effect of CNP on IgG leakage after HI insult and BMEC survival under OGD. Of importance, CNP significantly ameliorates brain atrophy and improves neurological deficits after HI insults. Altogether, the present study indicates that recombinant CNP exerts vascular protection in neonatal HI brain injury via its innate receptors, suggesting a potential therapeutic target for the treatment of neonatal HI brain injury.

## 1. Introduction 

Hypoxic-ischemic (HI) events such as birth asphyxia induce neonatal brain injury that initiates shortly after HI insults and develops over days [1,2]. With hypothermia as the only clinical therapeutic available, hypoxic-ischemic encephalopathy (HIE) remains a leading cause of death and disability in neonates. Previous studies revealed that blood–brain barrier (BBB) impairment occurs within hours after neonatal HI brain injury and may contribute to disease progression [3,4,5]. Multiple factors such as oxidative stress, nitrosative stress, and inflammation contribute to endothelial dysfunction and tight junction breakdown, which increase the risk of vasogenic edema and hemorrhage [6,7]. Lines of evidence have confirmed the presence of intracerebral hemorrhage and brain edema after HI brain injury [8,9,10]. Moreover, it is found that cerebral edema is closely associated with severe cases of HIE and worse neurological outcomes [4,10,11,12]. These findings suggest that BBB protection and brain edema reduction are potential strategies in the treatment of neonatal HI brain injury. 

C-type natriuretic peptide (CNP) is a potent neuropeptide released by vascular endothelial cells and neurons in various brain regions [13,14,15,16]. Besides its cardiovascular effects, mounting evidence documented a wide range of biological effects of CNP in the central nervous system. For instance, CNP was shown to increase cyclic guanosine monophosphate (cGMP) production in brain microvascular endothelial cells and astrocytes [17,18], inhibit astrocyte proliferation [19], regulate water channel aquaporin-4 expression [20], and promote axon bifurcation and neurogenesis [21,22]. Our previous study has demonstrated that CNP is an innate neuroprotectant after neonatal HI brain injury and preserves cortical neuron survival from oxygen-glucose deprivation (OGD) insults [23]. Given its widely accepted role in the vascular system and documented action in cellular components of the neurovascular unit, it is of great interest to determine the effect of CNP on the vascular damage following HI brain injury in the developing brain. 

Here, in the present study, using a neonatal HI brain injury model in mouse pups and an in vitro BBB model with primary brain microvascular endothelial cells, we explored the role of CNP in neonatal HI-induced cerebrovascular injury and the potential therapeutic effect of recombinant CNP in the treatment of neonatal HI brain injury. 

## 2. Results

### 2.1. Recombinant CNP Treatment Decreased Brain Infract, Cerebral Edema and IgG Leakage in Neonatal HI Brain Injury 

**Figure 1:**
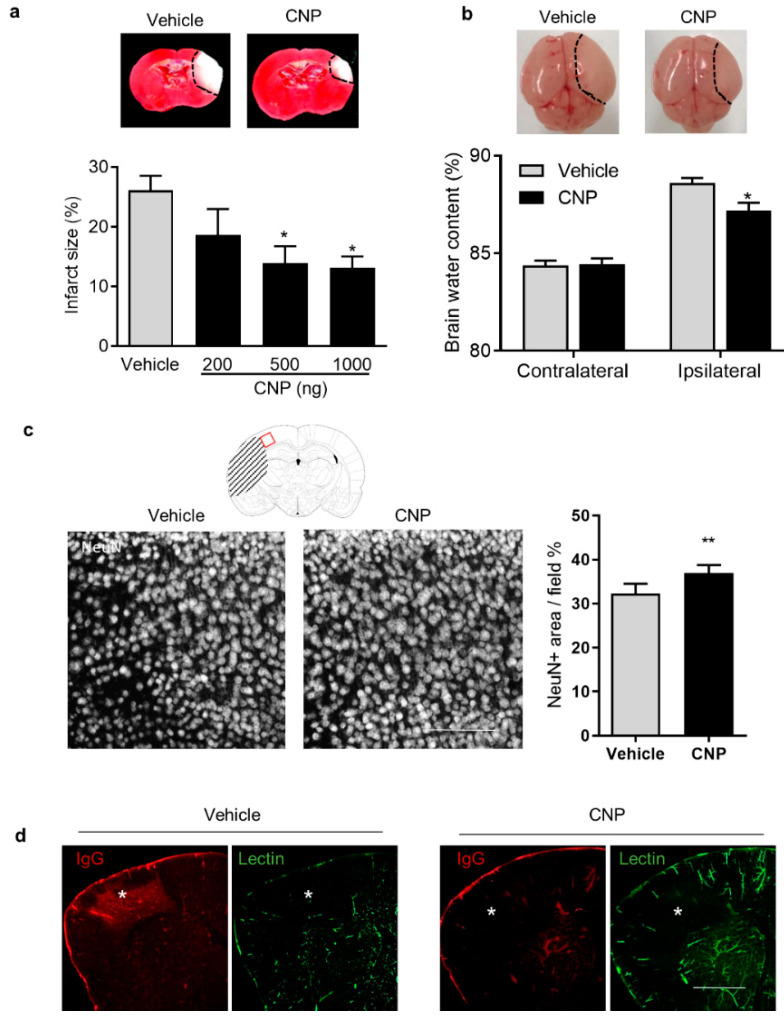
Recombinant CNP mitigates brain injury after neonatal hypoxic ischemic (HI) brain injury. (**a**) Representative images of TTC staining and quantification of brain infarct size 24 h after HI. N = 7–8 animals/group. *, *p* < 0.05 vs. Vehicle. (**b**) Representative images of brain edema and quantification of brain water content 24 h after HI. N = 6 animals/group. *, *p* < 0.05 vs. Ipsilateral in Vehicle. (**c**) Schematic diagram of the position of imaged area (red frame) and representative confocal images of NeuN staining in the target region of the vehicle and CNP treated brain 24 h after HI. Scale bar: 200 μm. Bar graph showing percentage of NeuN stained area per field, representing neuron density. N = 6–7 animals/group. **, *p* < 0.01 vs. Vehicle. (**d**) Representative confocal images showing IgG extravasation (red) and blood vessel density (lectin, green) in the ipsilateral hemisphere of the brain 24 h after HI. Asterisks indicate the area with decreased blood vessel density. Scale bar: 1000 μm.

To study the effect of CNP on neonatal HI brain injury, recombinant CNP with multiple doses (200, 500, and 1000 ng/pup) or PBS (Vehicle) was delivered into the ipsilateral hemisphere of P7 mouse pups via i.c.v. injection 2 h after HI insults. Twenty-four hours later, TTC staining was performed, and the result showed that CNP treatment with 500 and 1000 ng significantly reduced brain infarct size, compared with the Vehicle (Figure 1a). Thus, CNP treatment with 500 ng was used in the following experiments. At 24 h after HI insults, smaller visible brain swelling was developed on the ipsilateral hemisphere in CNP-treated pups, compared with the Vehicle (Figure 1b). Both contralateral and ipsilateral hemispheres were collected for brain water content assay. The result showed that CNP treatment significantly reduced brain water content in the ipsilateral hemisphere after HI insults, compare with the Vehicle group (CNP, 87.2% ± 0.40% vs. Vehicle, 88.6 ± 0.26%, *p* < 0.05, Figure 1b). Then, the coronal sections were immunostained to visualize neuronal loss and BBB permeability. The confocal images showed that CNP treatment significantly attenuated neuronal loss (Figure 1c) compared with Vehicle. In addition, reduction in IgG-positive regions and vascular loss (Lectin-positive staining) in the cortex of the ipsilateral hemisphere was observed in the CNP-treated brain, compared with the Vehicle (Figure 1d). 

### 2.2. Recombinant CNP Protected Brain Endothelial Cells from Oxygen–Glucose Deprivation Injury 

**Figure 2:**
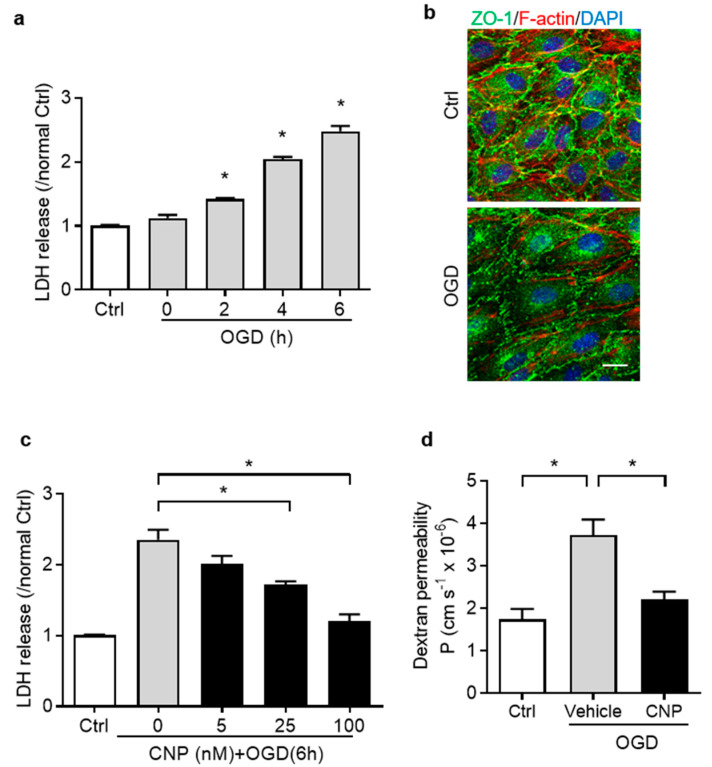
Oxygen–glucose deprivation (OGD) induces primary brain endothelial cell injury. (**a**) Lactate dehydrogenase (LDH) release from endothelial cells measured after 0, 2, 4 and 6 h of OGD treatment. Ctrl represents cells cultured without any treatments. N = 3–4 independent cell culture preparations. *, *p* < 0.05 vs. Ctrl. (**b**) Representative confocal images show the cytoskeleton (F-actin, red) and tight junctions (ZO-1, green) of endothelial monolayers treated with Ctrl or 6 h of OGD. Scale bar: 20 μm. (**c**) Endothelial cells were treated with 0, 25, 50 or 100 nM of CNP, and exposed to OGD for 6 h. LDH release was measured at the end of OGD treatment. (**d**) Dextran permeability coefficient of endothelial monolayer treated by vehicle or 100 nM CNP followed by 6 h of OGD. N = 3–4 independent cell culture preparations. In all panels, the control cells (Ctrl) are cultured under normoxia without any treatments *, *p* < 0.05.

We firstly established and optimized the OGD treatment model on endothelial cell viability by evaluation of the amount of lactate dehydrogenase (LDH) released in the culture medium. Endothelial cells were exposed to OGD for 0, 2, 4, and 6 h. The result revealed that OGD treatment significantly increased LDH release in a time-dependent manner (*p* < 0.05, Figure 2a). The OGD treatment for 6 h was selected for the following studies. To mimic BBB properties in vitro, endothelial cells were cultured to form a monolayer (>97% confluent), which showed a typical cobblestone pattern of ZO-1 tight junction protein and a cortical distribution of F-actin cytoskeleton by immunostaining [24]. When exposed to OGD for 6 h, endothelial cell monolayers were impaired showing reduced junction protein ZO-1 fluorescence intensity compared with Ctrl (Figure 2b). 

To examine the effect of recombinant CNP treatment on brain endothelial cell survival after OGD, multiple doses of recombinant CNP (0, 5, 25 or 100 nM) were added into the endothelial cell cultures for 4 h, followed by OGD insult. The result showed that CNP treatment dose-dependently decreased LDH release induced by OGD exposure (*p* < 0.05, Figure 2c). Additional results showed that CNP treatment (100 nM) didn‘t affect the LDH release compared to PBS under normoxia (Figure S1A). The barrier integrity of endothelial monolayers was examined by the measurement of FITC-conjugated dextran permeability at the end of OGD. The result showed that OGD exposure significantly increased the dextran permeability coefficient (*p* < 0.05, Figure 2d), indicating impaired barrier function. Incubation with CNP significantly decreased the dextran permeability compared with Vehicle (*p* < 0.05. Figure 2d). In addition, CNP treatment didn‘t affect the monolayer permeability to dextran by Dextran Permeability Assay under normoxia (Figure S1B).

### 2.3. Both NPR2 and NPR3 Were Involved in the Endothelial Protective Effect of CNP In Vitro

**Figure 3:**
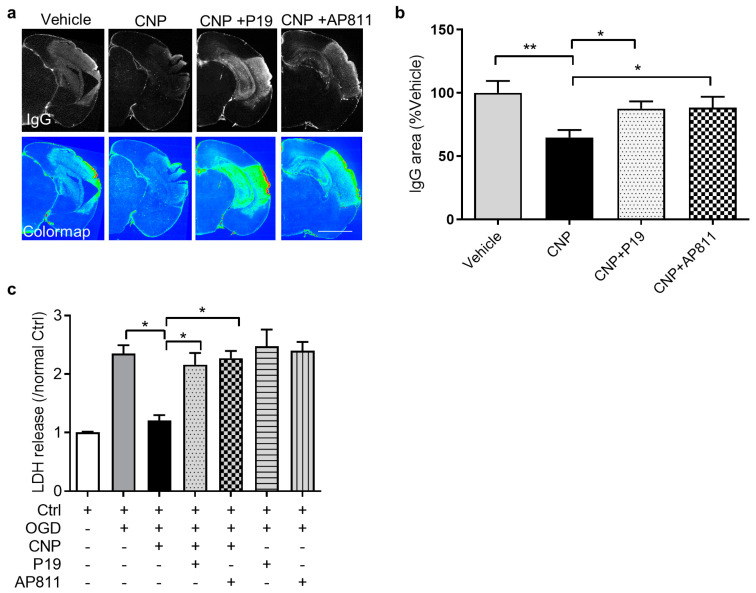
NPR2 and NPR3 mediate the vasculoprotective effect of CNP in vivo and in vitro. (**a**) Representative immunofluorescence images and colormap showing IgG extravasation in the brains of mice treated with PBS, CNP, CNP + P19 or CNP + AP811. Scale bar = 2 mm. (**b**) Quantification of the IgG extravasation area in ipsilateral hemispheres of mice from each group. The value is normalized by average area of leakage from animals treated with PBS. Three slices from each animal were used. N = 6–7 animals/group *, *p* < 0.05. (**c**) LDH release measured in endothelial cells subjected to indicate treatments after OGD treatment. n = 3–4 independent cell culture preparations. *, *p* < 0.05, **, *p* < 0.01.

The action of CNP is mediated by its cognate receptors NPR2 and NPR3. We further studied the role of NPR2 and NPR3 in the vasculoprotective effect of CNP in neonatal HI brain injury. Either NPR2 antagonist P19 or NPR3 antagonist AP811 was delivered into the brain of female and male mouse pups via i.c.v injection prior to HI brain injury followed by CNP treatment. Immunostaining of IgG was performed to measure BBB permeability 24 h after HI. The result showed that a strong IgG-positive signal was observed in the cortex of the ipsilateral hemisphere in Vehicle, which was reduced with CNP treatment. The IgG leakage in the groups of P19 or AP811 in combination with CNP was comparable to Vehicle, suggesting that the effect of CNP was countered by NPR2 or NPR3 antagonist (Figure 3a). The following quantification of the fluorescence density of IgG showed that CNP treatment significantly reduced IgG leakage into the brain tissue after HI, compared with Vehicle, which was reversed by P19 or AP811 treatment (Figure 3b). 

We next evaluated whether NPR2 and NPR3 mediated the cytoprotective effect of CNP on primary brain endothelial cells during OGD. Endothelial cells were treated with CNP alone or in combination with either P19 or AP811 followed by OGD treatment. At the end of OGD, the LDH release was detected. As predicted, either P19 or AP811 treatment significantly reversed the cytoprotective effect of CNP on endothelial cells after OGD, showing increased LDH release compared with CNP (Figure 3c). Inhibition of either NPR2 or NPR3 didn‘t affect the LDH release compared to PBS under normoxia. Together, these results suggest that the activation of both receptors is required for the vasculoprotective effect of CNP.

### 2.4. Recombinant CNP Reduced Cerebral Atrophy and Improved Neurological Outcome in Mice with Hypoxic-Ischemic Encephalopathy

**Figure 4:**
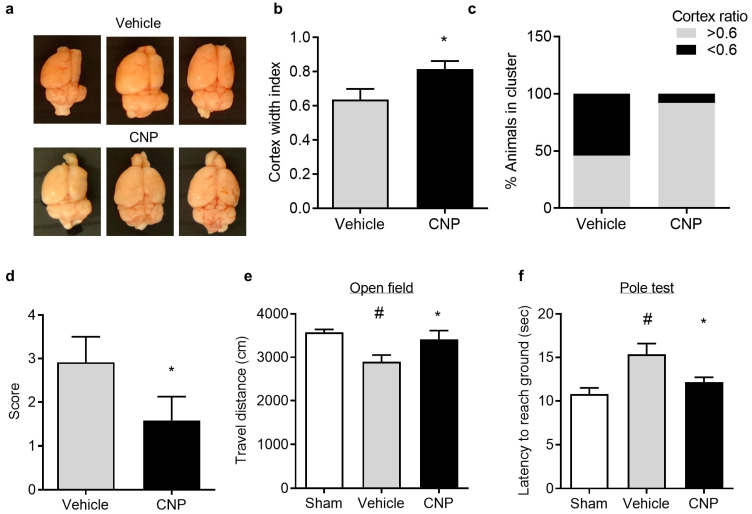
CNP treatment improves prognosis of HI brain injury in mice. (**a**) Representative images of brains show cerebral atrophy in vehicle or CNP treated mice after HI insults. (**b**) Quantification of brain atrophy by cortex ratio. Lower ratio indicates more tissue loss. (**c**) Pups in Vehicle- and CNP-treated group were separated into two clusters by a cortical ratio threshold of 0.6. Bar graph shows the percentage of mice in each cluster. (**d**) Neurological deficit score from Vehicle- or CNP-treated mice. Higher score indicates greater deficit. (**e**) Open field test shows locomotor activity of the animals in each group. (**f**) Results from a vertical pole test showing the average latency to descend from the pole to the ground for each group. N = 7−12 animals/group. #, *p* < 0.05 vs. Sham; *, *p* < 0.05 vs. Vehicle. All data was expressed as mean ± SEM.

To explore the long-term effect of CNP on neonatal HI brain injury, the cortical atrophy and neurobehavioral deficits were examined about four weeks after HI insults. The brain atrophy was evaluated by measurement of the ratio of ipsilateral hemisphere width to contralateral hemisphere width (the cortical ratio) (Figure 4a). The following quantification of cortical ratio showed that CNP treatment significantly attenuated the decrease in the cortical ratio after HI, compared with Vehicle (CNP 0.81 ± 0.05 vs. Vehicle 0.64 ± 0.06, *p* < 0.05) (Figure 4b). Moreover, 92% of pups in the CNP-treated group showed larger cortex ratio (>0.6) compared to only 46% in the vehicle-treated group (Figure 4c). In line with the reduced brain atrophy, CNP treatment also significantly improved neurological deficit score compared with Vehicle (*p* < 0.05, Figure 4d). In the open field test, animals showed a significantly reduced travel distance after HI insults compared with Sham, which was reversed by CNP treatment (*p* < 0.5, Figure 4e). In the vertical pole test, the latency of each mouse to descend from the top of the pole to the ground was measured. The result showed that the climbing down latency was significantly increased in animals after HI insults, compared with the Sham group, which was reduced in CNP-treated mice (Vehicle 15.4 ± 1.2 vs. CNP 12.17 ± 0.5 s, *p* < 0.05, Figure 4f). The neurobehavioral results suggest that CNP treatment can improve motor deficits induced by HI insults.

## 3. Discussion

CNP is the predominant natriuretic peptide present in the brain [25]. Our previous study demonstrated the innate neuroprotectant role of CNP in the developing brain after neonatal HI brain injury in mouse pups [23]. In the present study, we demonstrated that recombinant CNP protected the neonatal brain against HI-induced vascular damage. Furthermore, using primary brain endothelial cell cultures, we demonstrated that CNP preserved endothelial cell viability and endothelial cell monolayer barrier integrity from OGD insult. Moreover, the results from both in vivo and in vitro experiments revealed that the vasculoprotective effect of CNP was mediated by its innate receptors NPR2 and NPR3. Of importance, the recombinant CNP treatment mitigated brain atrophy and improved neurological functions after neonatal HI brain injury. Our findings indicate that CNP is beneficial for the immature brain after neonatal HI insults. 

BBB disruption is widely accepted as a potential therapeutic target for ischemic brain injury in adults, whereas it is less studied in neonatal HI brain injury. CNP is a potent neuropeptide released by vascular endothelial cells and neurons in various brain regions [15,16,26], and plays dual roles in neuronal survival [27] and vascular homeostasis maintenance [28]. Recent studies revealed that CNP is a vascular active peptide that possesses cardiovascular protective effects [29,30,31]. However, its function in cerebrovascular injury after neonatal HI brain injury remains unclear. We found that BBB disruption evidenced by brain edema and IgG extravasation was present in pups 24 h after HI insults. The recombinant CNP treatment significantly attenuated brain edema and BBB leakage in these mice. The BBB protective effect was further confirmed in brain endothelial cells in vitro in which CNP protected endothelial cells against OGD-induced cell injury and barrier breakdown. Under normoxia, the same doses of CNP did not change cell viability and barrier properties, indicating that the rescue effect of CNP to OGD insult is not a general phenomenon. These results are in line with previous studies in which CNP was shown to enhance the restoration of endothelial barrier function and attenuated endothelial activation after stress [32,33]. The BBB protection of CNP is likely associated with the capability of CNP to enhance endothelial survival rather than its regulatory capacity in BBB permeability, which may be bidirectional according to existing studies [16,34,35]. Thus, CNP may primarily mitigate pathological changes in brain vascular endothelial cells and preserve BBB integrity after neonatal HI brain injury. 

The action of CNP is mediated by its cognate receptors NPR2 and NPR3. RNA-sequencing data revealed that NPR2 is highly expressed in neurons and astrocytes with relatively lower expression in endothelial cells, while NPR3 is mainly expressed in endothelial cells [36]. NPR2 is a membrane guanylyl cyclase that is thought to mediate many of CNP’s biological actions [37]. In endothelial cells, the NPR2 activation by CNP increases cGMP [17,38,39], a second messenger that was shown to enhance endothelial survival and preserve barrier integrity after stress [40,41,42]. NPR3 is a Gi-coupled receptor without a guanylyl cyclase function [43,44]. NPR3 has been shown to promote endothelial proliferation [26,31], which may facilitate vascular recovery after HI. Its downstream signaling pathways such as PI3K/Akt [45] may provide vascular protection through restoration of endothelial nitric oxide synthase (eNOS) activity [46,47,48,49,50]. Indeed, we found that the vasculoprotective effect of recombinant CNP was countered by NPR2 or NPR3 antagonist. This result was further confirmed in brain endothelial cell cultures that antagonization of either NPR2 or NPR3 reversed the cytoprotective effect of CNP after OGD, suggesting the potential cooperative effect of NPR2 and NPR3 signaling pathways in endothelial cells in response to OGD. We expect that either NPR2 or NPR3 antagonist treatment will show increased LDH release after OGD compared to the Vehicle. However, in the results, neither P19 nor AP811 treatment exacerbates OGD-induced cytotoxicity on endothelial cells. Many factors may affect the outcome. One explanation for the phenomenon is that two receptors may show synergic effects in regulating endothelial cell viability under OGD conditions, and the receptor may partially compensate the inhibition of the other one. We also cannot rule out the potential influences of the dosage or the selectivity of the antagonists. Future studies are needed to further address this question. We have previously shown that NPR3 is not directly involved in cortical neuron survival after OGD [23]. The current results indicate that NPR3 exhibits indirect neuroprotection via vascular protection after neonatal HI brain injury. Though the molecular mechanism for CNP to protect endothelial cells after OGD is not yet known, the above-mentioned mechanisms may potentially contribute to the vasculoprotective effect of CNP. Future study should further investigate the CNP receptor-mediated signaling pathways in endothelial cell viability after OGD. 

In humans, up to 25% HIE survivors develop cerebral palsy and others may exhibit varying forms of disability, such as motor impairment, mental retardation and others [34,35,51]. In animal models of neonatal HIE, the primary focus of available studies is brain lesion [52]. Pups develop brain atrophy in response to the initial insult and prolonged time of recovery after HI exposure [53]. We found that mouse pups after HI brain injury exhibited brain atrophy four weeks after the initial insult, which was significantly diminished in the CNP-treated group. Moreover, a large proportion of CNP-treated mice showed smaller brain tissue loss compared with the Vehicle, suggesting the prolonged protective effect of CNP on HI brain injury. In agreement with the reduction in brain atrophy, recombinant CNP also provides long-term beneficial effects on locomotor function recovery after neonatal HI brain injury using neurological deficits scoring, open field test and vertical pole test [38,39]. The vertical pole test mainly reflects striatal lesion that has been shown to be present in mice after HI brain injury, especially those with more severe brain injury [54,55]. The results suggest that CNP treatment may potentially improve motor functions in severe HIE cases. 

## 4. Material and Methods

### 4.1. Animals and Surgical Procedures

Timed pregnant CD1 mice were purchased from Charles River Laboratories (Portage, MI, USA). The pregnant mice were housed under a 12-h light–dark cycle in the Animal Care Facility of Loma Linda University and had access to food and water ad libitum. All procedures and protocol were approved by the Institutional Animal Care and Use Committee of Loma Linda University and followed the guidelines by the National Institutes of Health Guide for the Care and Use of Laboratory Animals.

For all procedures that require surgery, induction and maintenance of anesthesia is achieved with 4% and 2% isoflurane in air, respectively. Loss of consciousness was confirmed by hind limb toe pinch. For euthanasia, mice were euthanized with 5% isoflurane followed by decapitation. To minimize pain and suffering of animals, post-operative ketoprofen (2 mg/kg, IM) was administered as necessary to relieve any signs of pain.

Neonatal HI brain injury was induced in mouse pups at postnatal day 7 (P7) under isoflurane anesthesia using a modified Rice–Vannucci model. Briefly, the right common carotid artery was dissected and ligated with an 8.0 silk surgical suture, and then cut between two ligation sites. The pups were allowed to recuperate for 1 h in a chamber warmed by an infrared lamp before being placed into hypoxia incubator supplied with humidified 8% oxygen balanced with 92% nitrogen for 15 min at 37 °C. At the end of hypoxia, pups were returned to their dams for recovery. All pups, including male and female, were used in the experiment. The overall sex ratio was approximately 1:1. 

### 4.2. Intracerebroventricular Injection

Animals were anesthetized with isoflurane and mounted on stereotaxic equipment. A total volume of 2 μL drug solution was stereotaxically injected into the ipsilateral hemisphere of mouse pups (placement coordinates: 0.8 mm lateral, 1.5 mm below the skull surface) with a flow rate of 0.5 μL/min. The needle was left in place for 2 min and slowly retracted. For the vehicle group, the same volume of PBS was injected into the mouse brains. The pups were allowed to recuperate for 15 min in a warmed chamber and returned to the dam. 

The stock solution of recombinant CNP (1-22, #3520, Tocris), NPR2 antagonist P19 (#00540, Phoenix pharmaceutical) and NPR3 antagonist AP811 (#5498, Tocris) was prepared in 0.1 M phosphate-buffered saline (PBS, pH 7.4) according to the manufacturer’s instruction. Either recombinant CNP (200, 500, or 1000 ng/2 μL) or vehicle (PBS) was delivered 2 h after HI brain injury via *i.c.v* injection. P19, AP811 (500 pmol/pup), or vehicle (PBS) was administered 4 h before HI brain injury via *i.c.v* injection followed by CNP treatment. 

### 4.3. Measurement of Brain Infarct Size

Brain infarct size was determined 24 h after HI using 2, 3, 5-triphenyltetrazolium chloride monohydrate (TTC, #T8877, Sigma-Aldrich, St Louis, MO, USA). The brains were harvested and dissected into coronal sections (2 mm thickness, 4 slices per brain), and immersed into pre-warmed 2% TTC in PBS for 5 min at 37 °C against light. Sections were washed with PBS and fixed in 10% formaldehyde overnight. The caudal and the rostral surfaces of each slice were scanned and the percentage of infarct area (average of both sides) in the ipsilateral hemisphere for each slice was traced and analyzed by the NIH Image J software (NIH, Bethesda, MD, USA). 

### 4.4. Brain Water Content Assessment

Brain water content was assessed twenty-four hours after the initial hypoxia incubation as described previously [56,57]. Pups were euthanized with 5% isoflurane and brains were harvested. The ipsilateral and contralateral hemispheres were separated and placed at 95 °C to dry. The percent water was calculated as (1-dry weight/wet weight) × 100%. 

### 4.5. Neurological Function Assessment and Brain Atrophy Assay

Neurobehavioral tests were performed about 4 weeks after HI insults. The neurological scoring criteria was adopted from methods as we previously described [58]. The pups were scored on a 3-point scale according to their performance in each trial. Two trials including forepaw extension and symmetry in limb movement were utilized. Pups were scored 2 points if there was a moderate to severe deficit in each trial, and no points if there was no visible deficit. In addition, the pups were also scored on their appearance including facial hair loss, eye abnormalities, and body size. Pups were scored 3 points if all three traits were observed. The assessment was carried out by a researcher who was blinded to group assignment. 

Vertical pole tests were used to test spontaneous activity and motor impairment, which was adopted from a previously reported method [38]. Briefly, a 65 cm vertical pole with a diameter of 8 mm was set up from the home cage. Animals were placed on top of the pole, and the time it took for the mice to climb down from the top of the pole to the home cage was recorded. 

The open field test was conducted in a box with dimensions 30 × 30 × 40 cm^3^; each mouse was placed in the arena and allowed to freely explore the box for 10 min. The process was recorded by an overhead camera and analyzed offline. The travel distance was calculated by analyzing the recorded video using NIH image J software with the MouBeAT plugin as previously reported [59,60]. 

In order to control bias during assessment, pups from different groups were housed together and arbitrarily picked for neurological function assessment. The person undertaking all measurements was blinded to group assignment. Mice were euthanized after a behavior test. The brains were fixed in 4% PFA, and the degree of cortical cavitation was measured using the previously validated ‘cortical width index’ [61,62]. Briefly, the images of the whole brain were taken, and the cortical width index was calculated as the distance from midline to the edge of the cavitation on the ischemic hemisphere divided by the distance from the midline to the lateral edge on the contralateral side.

### 4.6. Lectin Infusion and Immunofluorescence Staining 

Mice were anesthetized with isoflurane; retro-orbital injection of fluorescent labeled lectin (DL-1174, Vector laboratories) was performed 10 min before euthanasia. Brains were fixed in 4% PFA overnight, cryopreserved in 30% sucrose solution, embedded into Tissue-Tek optimal cutting temperature compound (OCT) (#23-730-571, Fisher Scientific) on dry ice, and cryosectioned at a thickness of 30 μm. For immunofluorescence staining, free float slices were blocked in PBS with 5% donkey serum (#017-000-121, Jackson ImmunoResearch) and 0.3% Triton X-100 for 1 h, then incubated with Primary antibody NeuN (1:500, #ab177487, Abcam) in PBS with 1% donkey serum and 0.3% Triton X-100 overnight at 4 °C. After washing 3 times with PBS, slices were incubated with fluorescence-labeled secondary antibodies (1:500, #A31570, Invitrogen) for 1 h at room temperature, then mounted on glass slides, cover-slipped with mounting medium (#S36939, Life technology), and imaged on a Zeiss LSM 710 NLO confocal microscope (Zeiss, Oberkochen, Germany). The Neural density in the peri-infarct region of each mouse was assessed as previously described [63,64]. Briefly, the coronal sections from bregma 1-3 mm were used. Images of the NeuN immunofluorescent staining in the peri-infarct region were collected. The percentage of NeuN staining under each field of view were determined using NIH Image J software (NIH, Bethesda, MD, USA). For the assessment of immunoglobulin G (IgG) extravasation, slices were blocked and incubated with fluorescence labeled anti-mouse IgG secondary antibody (1:500, Invitrogen, CA , USA) for 2 h at room temperature. Nuclei were stained by Hoechst. After mounting and coverslip, slices were tile scanned on EVOS^®^ FL Auto Imaging System (Life Technologies, Carlsbad, CA, USA). Monochromic images of IgG fluorescence were converted to colormap using python3 opencv package. Original code is available upon request. Slices at 1-3 mm posterior to bregma from each animal were used for quantification. The area of IgG positive regions was measured using NIH Image J software (NIH, USA). 

### 4.7. Oxygen–Glucose Deprivation (OGD) and Lactate Dehydrogenase (LDH) Assay

Primary human brain microvascular endothelial cells (HBMEC, #1000, ScienCells, Hongkong, China) were subjected to an oxygen–glucose deprivation (OGD) procedure adopted from previous studies [65,66]. Briefly, endothelial cells were cultured in endothelial cell medium with endothelial cell growth supplement (#1001, ScienCell, Carlsbad, CA, USA) on collagen-coated culture plates. The culture media were replaced with pre-warmed no-glucose DMEM (Gibco) pre-equilibrated with 1% oxygen. Cells were incubated in a humidified hypoxia chamber with 1% oxygen and 5% carbon dioxide at 37 °C for the required period of time. Cells cultured under normal conditions were used as a control (Ctrl). For the endothelial cells protective effect study, cells were incubated with CNP at concentration of 0, 5, 25, 100 nM. To study the role of natriuretic peptide receptors (NPRs), HBMECs were incubated with NPR2 antagonist P19 (100 nM) or NPR3 antagonist AP811 (100 nM) alone, or with the presence of CNP (100 nM).

The degree of cell viability at the end of OGD was assessed by detecting the amount of lactate dehydrogenase (LDH) released in the culture medium using CyQUANT™ LDH Cytotoxicity assay kit (#C20300, Fisher Scientific, Hampton, NH, USA) according to the manufacturer’s instructions. The background signal (absorbance at 630 nm) was subtracted from reaction signal (absorbance at 490 nm). LDH release was normalized to protein concentration and the results were shown as fold changes over normal controls, as we described previously [23,67]. For immunostaining, cells were seeded into a 24-well plate with collagen coated coverslips. After assigned cell culture procedure, cells were fixed in 4% PFA for 30 min. After washing away the PFA, immunostaining was carried out as described above. 

### 4.8. Dextran Permeability Assessment 

Dextran permeability assessment was performed at the end of OGD insult as previously described [24]. Briefly, endothelial cell monolayer of full confluency was produced by plating 5 × 10^5^ cells/cm^2^ in the upper chamber of tissue culture inserts pre-coated with rat tail collagen I (Corning, Coring, NY, USA). Cells were cultured in Endogrow media for 2 days. Fluorescent labeled dextran (40 kDa) was added to the insert well. The permeability coefficient was calculated as reported [24]. Briefly, the volume cleared (ΔVc) was calculated as ΔVc = C_lower_ × V_lower_/C_upper_, where C_upper_ and C_lower_ are FITC-labeled dextran concentrations in upper and lower chambers, respectively, and V_lower_ is the volume in lower chamber. The volume cleared (ΔVc) was plotted against time, and the permeability surface area (PS) product was obtained from the slope by linear regression. The permeability coefficient (P) was then calculated by *P* = PS/s, where s is the surface area of the filter. Finally, the permeability coefficient of cells (P_cell_) was obtained by correcting the overall permeability coefficient (P_cell+filter_) for that of the cell-free filter (P_filter_) using 1/P_cell_ = 1/P_cell+filter_ − 1/P_filter_. P_filter_ was determined on a separate series of experiments using the cell-free filter inserts only.

### 4.9. Statistical Analysis 

All data was expressed as mean ± standard error of the mean (SEM), plotted and analyzed in GraphPad Prism Software (San Diego, SD, USA). A normality test was performed on residuals using a Shapiro–Wilk test and Kolmogorov–Smirnov test in SPSS 26 (IBM, Armonk, NY, USA). For data that pass the normality test, comparisons between two groups were analyzed using the Student’s *t* test, and multiple comparisons were analyzed using one-way ANOVA followed by Newman–Keuls post hoc test. For datasets that do not pass the normality test, a nonparametric test was used. A *p* value of less than 0.05 was considered significant. 

## 5. Conclusions 

In conclusion, the present study provides novel evidence of the vasculoprotective effect of CNP in the neonatal brain after HI insults. Recombinant CNP treatment reduced brain edema and IgG leakage into the brain tissue induced by HI insults, which is further validated using an in vitro OGD model in cultured primary brain endothelial cells. Moreover, CNP treatment provides a prolonged neuroprotective effect by mitigating brain atrophy and improves neurological functions in mice after neonatal HI brain injury. Taken together, this study deepens our understanding of the role of CNP and its receptors in HI-induced cerebrovascular injury in the neonatal brain, which represents a potential therapeutic target for the treatment of neonatal HI brain injury.

## Data Availability

The data presented in this study are available in article/supplementary material.

## Supplementary Materials

The following are available online at https://www.mdpi.com/article/10.3390/ijms22168966/s1.

## Abbreviations

BBB	blood–brain barrier
CNP	C-type natriuretic peptide
HI	hypoxic ischemic
HIE	hypoxic-ischemic encephalopathy
IgG	immunoglobin G
LDH	lactate dehydrogenase
NPR2	natriuretic peptide receptor 2
NPR3	natriuretic peptide receptor 3
OGD	oxygen glucose deprivation
TTC	2,3,5-triphenyltetrazolium chloride
ZO-1	Zonula occludens-1
