# Supplementary material for: C-Type Natriuretic Peptide Ameliorates Vascular Injury and Improves Neurological Outcomes in Neonatal Hypoxic-Ischemic Brain Injury in Mice
Source: Int J Mol Sci. 2021 Aug 20;22(16):8966. doi: 10.3390/ijms22168966 (PMC8396645; doi:10.3390/ijms22168966)
Supplement: Supplementary file 1 [file ijms-22-08966-s001.zip › ijms-1332583-SI.pdf]

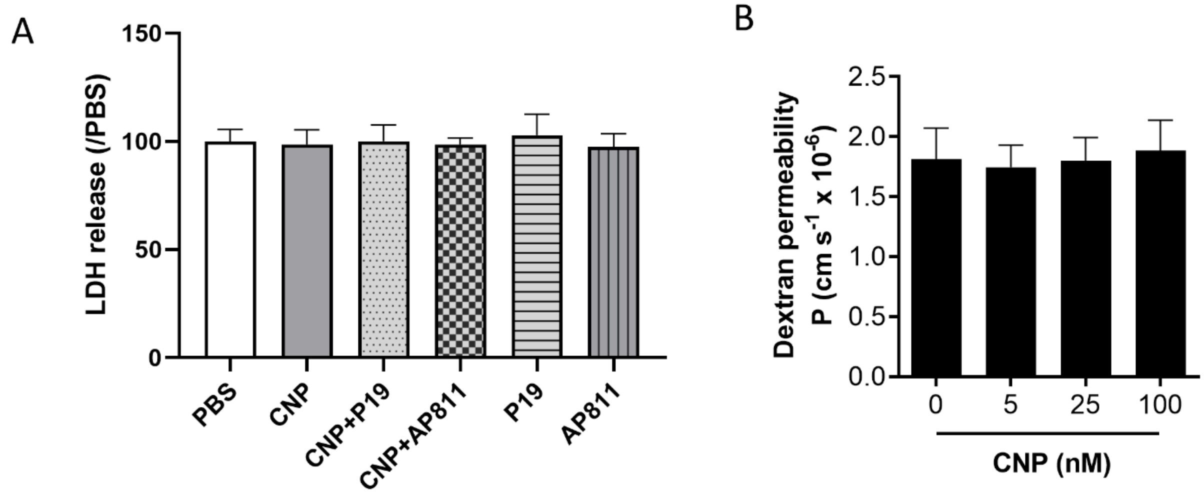

Figure S1. Control experiments under normoxia. A) LDH release measured in microvascular endothelial cells subjected to treatments without OGD. All drugs have no cytotoxic effect on those cells. n=5-6. B) Dextran permeability coefficient of endothelial monolayer treated by 0, 25, 50 or 100 nM of CNP without OGD. n=3. CNP did not significantly change the monolayer property measured by Dextran Permeability Assay.
